# DNA Triplet Energies by Free Energy Perturbation Theory

**DOI:** 10.1021/acs.jctc.4c01583

**Published:** 2025-01-24

**Authors:** Rafael García-Messeguer, Miriam Navarrete-Miguel, Sergio Martí, Iñaki Tuñón, Daniel Roca-Sanjuán

**Affiliations:** † Instituto de Ciencia Molecular, 201469 Universitat de València, 22085 València, Spain; ‡ Departament de Química Física i Analítica, Universitat Jaume I, 12071 Castelló, Spain; § Departamento de Química Física, Universitat de València, C/Dr. Moliner 50, 46100 Burjassot, Spain

## Abstract

Determining the energetics of triplet electronic states of nucleobases in the biological macromolecular environment of nucleic acids is essential for an accurate description of the mechanism of photosensitization and the design of drugs for cancer treatment. In this work, we aim at developing a methodological approach to obtain accurate free energies of triplets in DNA beyond the state of the art, able to reproduce the decrease of triplet energies measured experimentally for *T* in DNA (270 kJ/mol) vs in the isolated nucleotide in aqueous solution (310 kJ/mol). For such purposes, we adapt the free energy perturbation method to compute the free energy related to the transformation of a pure singlet state into a pure triplet state via “alchemical” intermediates with mixed singlet–triplet nature. By this means, standard deviation errors are only a few kJ/mol, contrary to the large errors of tenths of kJ/mol obtained by averaging the singlet and triplet energies derived from molecular dynamics simulations. The reduced statistical errors obtained by the free energy perturbation approach allow us to rationalize with confidence the triplet stabilization observed experimentally when comparing the thymine nucleotide and thymine in DNA. Spin polarization rather than excimer interactions between the π-stacked nucleobases originates the lower values of the triplet energies in DNA. The developed approach implemented in QM^3^ shall be useful for determining free energies of triplets and other states like ionic or charge separation states in any other macromolecular system with impact in biomedicine and materials science.

## Graphical abstract

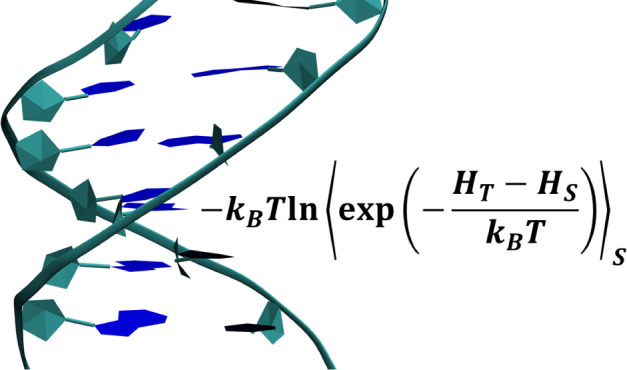

## Introduction

Triplet electronic states of nucleobases are key to the understanding of DNA damage by photosensitization and its application in the treatment of cancer.^1^ In this process, UVA light is absorbed by an exogenous or endogenous agent with a high quantum yield for triplet population (so-called photosensitizer). A triplet excited state is subsequently formed, with a longer lifetime than the singlet, which next transfers the excess energy to the nucleobases. Long-distance (Förster) or short-distance (Dexter) energy transfer mechanisms are possible for singlets, while only the latter applies for triplet–triplet energy transfer (TTET).^2,3^ Experimental studies on the yield of cyclobutane pyrimidine dimers (CPD) production in a DNA chain with covalently bonded benzophenone as a photosensitizer showed that Dexter TTET is facilitated by the stacking arrangements of nucleobases.^4^ This transfer is even more favorable when the photosensitizer and nucleobase molecules form an adjacent π-stacked exciplex complex. As proved by molecular dynamics simulations, the intercalation of benzophenone between two base pairs of the double helix is a favorable binding process.^5^ Highly accurate quantum chemistry also demonstrated, as experiments did, that benzophenone intercalated in the DNA can efficiently transfer the energy (TTET) to thymine.^5^

One relevant aspect in DNA photosensitization by the transfer of the triplet energy is that the triplet energy of the photosensitizer must be higher than that of nucleobases. In this context, experiments combining data obtained by spectroscopy and electrophoresis assays with agarose gels and using distinct photosensitizers allowed for providing an approximate value of 270 kJ/mol for thymine in a supercoiled circular DNA (pBR322).^6,7^ This is lower than the triplet energy measured for the thymidine 5′-monophosphate (TMP) nucleotide in both unbuffered and buffered aqueous solutions, with negligible changes when going from base to nucleoside to nucleotide (310 kJ/mol).^6,7^ Note, however, that the 270 kJ/mol value is an average number corresponding to many DNA sequences and distinct environments of thymine occurring in the supercoiled circular DNA. Hence, the source of the different triplet values in the nucleotide and the nucleic acid is not clear. In DNA, thermal fluctuations and interactions with the environment affect the triplet energies. In the isolated dimers, the decrease in triplet energy is proved theoretically to be associated with excimer interactions.^8^ This could explain the low experimental value determined in the supercoiled circular DNA. However, the backbone geometrical constraints do not allow for a perfect parallel configuration between adjacent nucleobases that is required for significant excimer binding through π-stacking.

Computational chemistry offers strategies to estimate triplet energies in macromolecular environments such as nucleic acids. Note that we focus herein on adiabatic singlet-triplet excitation energies (or simply adiabatic triplet energies), which correspond to the energetic difference between the equilibrated triplet excited state and the equilibrated singlet ground state. The reason is that intramolecular vibrational relaxation is typically faster (subpicosecond time scales) compared to TTET. One of the strategies that can be used to estimate the adiabatic triplet energies is to first run a molecular dynamics (MD)^9^ simulation of DNA, next carry out quantum mechanics/molecular mechanics (QM/MM)^10^ geometry optimization of the singlet ground state and triplet excited state of the nucleobase of interest in several conformations from the MD, and finally obtain the mean value of the singlet–triplet energy differences. The disadvantage of this strategy is that it does not consider the effect of the triplet electronic structure on the conformational dynamics of the environment. Furthermore, it does not consider the thermal energies because the geometry optimization brings the structure of the QM region to the minimum of the potential energy hypersurface. Therefore, the estimated triplet value is not strictly comparable with the experimental counterpart. Another approach is to perform two QM/MM MD simulations, one for the singlet ground state and another for the triplet excited state, and then take the difference between the mean value of the energies from each set of MD conformations (or the mean value of the singlet–triplet energy differences). In such a manner, triplet energies are computed as the internal energy change (Δ*U*) between the triplet and singlet states. This approach was used in a previous work, obtaining triplet energies of 305–329 kJ/mol when a single thymine molecule is considered in the QM part (therefore not allowing triplet excimer formation) and 300 kJ/mol when considering 4 adjacent thymine nucleobases (allowing in this case the possibility of forming triplet excimers). However, large standard deviation (SD) errors were obtained in such a study (∼50 kJ/mol for the computations with the single QM thymine nucleobase and 100 kJ/mol for the model with 4 QM thymine bases).^11^ These large errors are inherent to such a strategy, and are derived from the large noise of the MM part in the QM/MM MD simulations. Since they are as large as the energy difference obtained experimentally for the triplet energy of thymine in the isolated nucleotide and in the double helix, it makes it difficult to rationalize the computed values obtained for different conditions (solvent, DNA, etc.).

Hence, in this work, we aimed at developing a computational approach to obtain highly accurate energies of triplets in DNA beyond the state of the art and with minimal SD able to reproduce with accuracy the decrease of the triplet energies measured experimentally for thymine in DNA vs in the isolated nucleotide.^6^ To minimize the SD and get accurate energies, we attempted to compute directly the free energy change (Δ*G*) in going from the singlet to the triplet by adapting the technique based on alchemical free energy perturbation^12^ (and related to umbrella sampling^13^ or string method).^14^ Since in this strategy the MM component remains consistent across both states, the noise from this part is minimized. Therefore, this method allows for an accurate calculation of the energetics of the triplet state in a macromolecular environment with minimal standard deviation, which we anticipate to be only a few kJ mol^–1^. This new approach is not only applicable to triplet energy calculations but also holds potential for accurately determining other properties, such as ionization and charge separation in macromolecules relevant to biomedicine and materials science.

## Methodology

### Free Energy Perturbation Adapted for Spin Conversions

The free energy perturbation (FEP) method, as introduced by Robert W. Zwanzig in 1954,^15^ uses statistical mechanics and molecular dynamics or Monte Carlo simulations to compute free energy differences between two states. On the basis of the Zwanzig equation (eq 1), the Gibbs free energy differences for converting a state A into a state B (Δ*G*), which in condensed phases are numerically equivalent to Helmholtz free energy differences (Δ*F*), can be computed as

1 $$\Delta G\left(A\to B\right)\approx \Delta F\left(A\to B\right)=F_{B}-F_{A}=-k_{B}T\ln\langle \exp{\left(-\frac{H_{B}-H_{A}}{k_{B}T})\right\rangle }_{A}$$

where *k*_B_ is the Boltzmann constant, *T* is temperature, and *H*_A_ and *H*_B_ are the Hamiltonian energies of states *A* and *B*, respectively. The angular brackets denote an average over a simulation performed for state *A*. Therefore, for each conformation obtained in the simulation of *A*, a computation of the energy of state *B* is also carried out to obtain the energy difference between *B* and *A*.

Our initial hypothesis for obtaining triplet free energies in macromolecules is that we can create a number of intermediate alchemical (unphysical) states (or windows) for which their energies are obtained by a perturbation parameter λ and the associated Hamiltonian:

2 $$H\left(\lambda \right)=\left(1-\lambda \right)H_{S}+\lambda H_{T}$$

where *H*_S_ represents the initial ground singlet state (λ = 0) and *H*_T_ stands for the final triplet state (λ = 1). Free energies can then be calculated by the eq 3:

3 $$\Delta G=\overset{N-1}{\underset{i}{\sum }}\Delta G_{i,i+1}=-k_{B}T\overset{N-1}{\underset{i}{\sum }}\ln\langle \exp{\left(-\frac{H_{i+1}-H_{i}}{k_{B}T})\right\rangle }_{i}$$

in which for each window, the free energy difference Δ*G*_*i*,*i*+1_ between the two states *i* and *i*+1 is computed by averaging the energy difference *H*_*i*+1_ – *H*_i_ evaluated over a set of configurations of *i*. The error of the free energy difference (ε) can be easily evaluated by means of a first-order expansion, as proposed by Chipot,^16^

4 $$\varepsilon \left(\Delta G\equiv x\right)\approx \pm k_{B}T\frac{\delta \varepsilon }{{\left\langle e^{-\left(H_{j}-H_{i}\right)/k_{B}T}\right\rangle }_{i}}$$

5 $$\delta \varepsilon^{2}=\frac{1+2\tau }{N}\left[{\left\langle e^{-\left(H_{j}-H_{i}\right)/k_{B}T}\right\rangle }_{i}-{\left\langle e^{-\left(H_{j}-H_{i}\right)/k_{B}T}\right\rangle }_{i}^{2}\right]$$

6 $$1+2\tau =\frac{1+r_{1}}{1-r_{1}}$$

7 $$r_{1}=\frac{\sum \left(x_{i}-\mathrm{x̅}\right)\left(x_{i-1}-\mathrm{x̅}\right)}{\sum {\left(x_{i}-\mathrm{x̅}\right)}^{2}}$$

where δε is the statistical error of the ensemble average ${\left\langle e^{-\left(H_{j}-H_{i}\right)/k_{B}T}\right\rangle }_{i}$ , *N* stands for the number of points in each ensemble average, and 1 + 2τ is the sampling ratio, where τ corresponds to the correlation length.^17^

### Computational Details

To obtain the structures needed for calculating the FEP, we conducted hybrid QM/MM^10^ MD^9^ simulations using the implementation that we have carried out in the QMCube (QM^3^) program.^18^ The MM part was simulated using the AMBER DNA OL-15 force field,^19–21^ with the exception of the water molecules, which were modeled using the TIP3P force field.^22^ We used for the treatment of the QM part various methodologies, including semiempirical, density functional theory (DFT)^23^ and multi-state complete-active-space second-order Perturbation Theory (MS-CASPT2)^24^ methods, as further detailed.

Three types of systems were considered in this work: isolated thymine, thymidine 5′-monophosphate (TMP) in water solution, and a DNA helix formed by 10 thymine-adenine base pairs. Isolated thymine was used for benchmarking purposes of QM methods and comparisons with previous works. In the second system, TMP was introduced inside a water cubic box of 50 Å side. The QM part was the nucleobase. In the nucleic acid system, the DNA helix was embedded in an octahedral water box with a buffer of 12 Å around the DNA strand, comprising 3044 water molecules and 18 sodium atoms (needed to neutralize the total charge), resulting in a total of 9788 atoms. Distinct QM parts were used, comprising from 1 to 4 adjacent thymine nucleobases. The link atoms were located on the dangling N-glycosidic bond that connects the sugar–phosphate backbone with the nitrogenous base.

To prepare representative initial structures of the TMP and DNA systems, 100 ns classical MD simulations at 300 K were carried out with the AMBER program.^19,20^ Periodic boundary conditions were used with the cubic and the octahedral water boxes for the TMP and DNA models, respectively. Next, by using the QM^3^ implemented algorithm, we conducted QM/MM MD simulations for 100 ps of simulation time. In the TMP system, every atom further away than 18 Å from the nucleotide was frozen. For the DNA system, we froze every atom further away than 16 Å from the middle thymine nucleobase. QM/MM MD simulations were done for the pure singlet state (λ = 0), the pure triplet state (λ = 1), and 4 “alchemical” intermediates between the singlet and triplet states (λ = 0.2, 0.4, 0.6, and 0.8). To simulate those intermediates at each step of the simulation, the orbitals of the QM part were calculated in the singlet and triplet states. Then, depending on the intermediate, the energy and energy gradient were calculated as a weighted linear combination of the two states (see eq 2). The methods used for the simulations were geometries, frequencies, and noncovalent interactions–extended tight-binding (GFN2-xTB),^25^ hereafter referred to as xTB, and DFT with the hybrid Becke 3-parameter Lee–Yang–Parr (B3LYP)^26,27^ functional and the 6-31G* basis set. Hereafter, we shall call these simulations QM­(xTB)/MM MD and QM­(B3LYP)/MM MD, respectively. The former were carried out with QM^3^ interfacing xTB and fDynamo^28^ and the latter with QM^3^ interfacing Gaussian16 ^29^ and fDynamo.

Free energy differences obtained using the xTB and B3LYP methods were also improved by performing MS-CASPT2 energy computations on top of xTB and DFT geometries [QM­(CASPT2//xTB)/MM MD and QM­(CASPT2//B3LYP)/MM MD levels, respectively]. The basis set used was ANO-L-VDZP.^30–32^ The charges of the MM atoms were considered as an external field perturbing the MS-CASPT2 wave function. The active space used was 8 electrons in 10 orbitals for one nucleobase and 12 electrons in 12 orbitals for two nucleobases, comprising the π and π* space. Two roots for both the singlets and the triplets were used for the calculations. The zeroth-order Hamiltonian with an Ionization-Potential Electron-Affinity (IPEA)^33^ value of 0.25 and an imaginary level shift^34^ of 0.2 au was used. These multiconfigurational quantum chemistry computations were carried out with the OpenMolcas program.^35,36^

For the calculations of the triplet energies, we computed both Δ*U* and Δ*G* with the aim of conducting a comparison between them. The former is calculated as the difference between the average energy of 100 conformations obtained from the QM/MM MD in the singlet state (λ = 0) and the same number for the triplet state (λ = 1). The error corresponds to the SD of the data. On the contrary, Δ*G* ± ε is computed with the adapted FEP approach as described in the previous section.

## Results and Discussion

### Methodology Benchmarks for the Isolated Thymine Molecule

In order to choose a method with a good balance between accuracy and computational cost for the QM/MM MD simulations, the geometries of both the singlet ground state and triplet excited state of the isolated thymine were determined with the semiempirical xTB, Austin model 1 (AM1),^37^ parametrized model 3 (PM3),^38^ and parametrized model 6 (PM6)^39^ methods; and DFT with the hybrid meta-GGA Minnesota 06 with 54% of Hartree–Fock exchange (M06-2X)^40^ and B3LYP^26,27^ functionals. Table 1 compiles the results obtained regarding the energy differences between the triplet and singlet states. Energy values from the literature obtained with the multiconfigurational CASPT2 method and the high-accuracy coupled cluster singles, doubles, and triplets perturbatively added (CCSD­(T)) method were also included in Table 1. As can be seen, AM1, PM3, and PM6 largely stabilize the triplet states, giving rise to deviations higher than 1 eV. Meanwhile, xTB, M06-2X, and B3LYP show a better agreement as compared to CASPT2 and CCSD­(T). From these methods, we shall choose the xTB and B3LYP for the QM/MM MD simulations, the former because of its markedly reduced computational cost despite being semiempirical, and B3LYP for its good performance while keeping a lower cost as compared to M06-2X.

**Table 1:** Triplet Electronic Energies (Δ*E*, in kJ mol^–1^) in the Isolated Thymine Nucleobase Computed Herein and from the Literature with different Quantum Chemistry Methods and Error Relative to the CASPT2 Result

	Δ*E*	error (%)
AM1	144	–48.0
PM3	137	–50.5
PM6	142	–48.7
xTB	329	18.8
M06-2X	295	6.5
B3LYP	290	4.7
CASPT2//CASSCF(14,10)/ANO-S C,N,O[3s2p1d]/H[2s1p]^a^	277	0.0
CCSD(T)/6-31G(d,p)^b^	296	6.9
CCSD(T)/6–311++G(d,p)^b^	288	4.0

a Datum from ref. 41.

b Data from ref. 42.

### Triplet Free Energies of Thymidine Nucleotide in Water

Table 2 compiles the Δ*U* and Δ*G* data obtained for TMP in water corresponding to the QM­(xTB)/MM MD and QM­(B3LYP)/MM MD simulations. Data for the FEP approach with energy corrections at the CASPT2 level are also shown (CASPT2//xTB and CASPT2//B3LYP). Initially, we can observe that Δ*U* energies of thymine triplets in TMP yield a significant statistical error, in agreement with the findings by Allahkaram, Monari, and Dumont.^11^ By employing the new methodology, FEP, and computing the Δ*G*, a substantial reduction in this error occurs by 2 orders of magnitude for both xTB and B3LYP, giving rise to a more precise prediction. This is attributed to the cancellations of large fluctuations from the MM environment.

**Table 2:** Triplet Internal Energies (Δ*U*) and Free Energies (Δ*G*), in kJ mol^–1^, and the Corresponding Errors Computed for TMP in Water Solution by using different Statistical Approaches and Methods^a^

	Δ*U*	Δ*G*
xTB	291 ± 36	317.7 ± 0.8
B3LYP	201 ± 49	271.3 ± 0.1
CASPT2//xTB		325 ± 2
CASPT2//B3LYP		315 ± 1

a The Corresponding Experimental Value is 310 kJ mol^–1^.^6,7,43^

We shall now compare the obtained FEP values with the experimental value of 310 kJ/mol for TMP in aqueous solution.^43^ As observed, the energy obtained with the xTB method aligns well with the experimental value, while DFT/B3LYP underestimates this triplet energy value. When employing the CASPT2 method on top of the xTB and B3LYP geometries, the energy data increase and become closer. The xTB, CASPT2//xTB, and CASPT2//B3LYP give data reasonably consistent with the reference experimental energy.

### Triplet Free Energies of Thymine in DNA

Data related to the DNA systems are compiled in Table 3. As we did for TMP, it is worth comparing Δ*U* and Δ*G* results obtained with QM­(xTB)/MM MD and QM­(B3LYP)/MM MD simulations and from the CASPT2//xTB and CASPT2//B3LYP corrections. Furthermore, we include in the table data computed using a distinct number of thymine molecules in the QM part of the QM/MM MD simulations, which should be interesting to analyze quantum π-stacking and delocalization effects.

**Table 3:** Triplet Internal Energies (Δ*U*) and Free Energies (Δ*G*), in kJ mol^–1^, and the Corresponding Errors Computed for Thymine Nucleobase in DNA by using 1, 2, 3, and 4 Nucleobases in the QM Part and with different Statistical Approaches and Methods (see text)

method	Δ*U*	Δ*G*
QM (1 T)		
xTB	300 ± 37	320.1 ± 0.1
B3LYP	235 ± 38	274.0 ± 0.2
CASPT2//xTB		330 ± 2
CASPT2//B3LYP		319 ± 4
QM (2 T)		
xTB	270 ± 37	312.5 ± 0.1
B3LYP	229 ± 48	275.3 ± 0.5
CASPT2//xTB		322 ± 2
CASPT2//B3LYP		306 ± 3
QM (3 T)		
xTB		293 ± 4
QM (4 T)		
xTB		289 ± 5

The first observation that can be made by analyzing the data in Table 3 is related to the differences between the QM methods and is the same as for the analyses related to TMP: QM­(B3LYP)/MM MD gives rise to lower energies compared to the QM­(xTB)/MM MD. Note that even though the QM­(B3LYP)/MM MD value (Δ*G*) approaches the experimental energy (270 kJ mol^–1^),^6,7^ we must not attempt to match this value since the experiment corresponds to an average of several unknown DNA sequences and double helix conformations, while our model system is a poly-A–T sequence. As can be seen in Table 3, the QM­(B3LYP)/MM MD value is also clearly lower than the higher-level energy-corrected CASPT2//xTB or CASPT2//B3LYP triplet energies. Hence, we conclude that the QM­(B3LYP)/MM MD approach used here underestimates the triplet energies.

Another observation, also detected for TMP, is the difference regarding the estimation of triplet energies as Δ*U* or as Δ*G*: the latter decreases the statistical error by 2 orders of magnitude. This higher accuracy achieved with the determination of Δ*G* shall allow, herein for the first time, the detection of whether there is physical meaning behind the change in the triplet energies between the nucleotide and the DNA environments. As already mentioned in the Introduction, this is not possible by using the standard approach to determine triplet energies (as Δ*U*) because the standard deviations are so high that there is no statistically significant difference.

The xTB method was used herein for obtaining QM/MM MD energies with an increasing number of π-stacked thymine nucleobases, from 1 to 4. We used xTB because it better agrees with the CASPT2 energy-corrected values and is affordable for larger molecular size structures as compared to the other methods. As can be seen, upon increasing the number of “quantum” nucleobases, the computed Δ*G* (FEP) decreases, reaching a converged value of 289 ± 5 kJ mol^–1^ for 4 QM thymine nucleobases, which only differs by 4 ± 9 kJ mol^–1^ from the simulation with 3 QM bases. This stabilization of the triplet thymine bases in DNA is also observed for the reference CASPT2 method by comparing 1 and 2 QM nucleobases. DFT/B3LYP does not capture such a decrease, which must be attributed in part to the lack of dispersion corrections in the functional. When CASPT2 corrections are added on top of the DFT/B3LYP results, the triplet stabilization is again obtained.

Our best estimation of triplet energy in DNA, obtained at the QM­(xTB, 4 T)/MM MD level and using the developed FEP approach (289 ± 5 kJ mol^–1^) can now be used to interpret the experimental measurements of triplet energies in TMP and DNA (310 vs 270 kJ mol^–1^, respectively).^6,7,43^ As already stated above, our goal is to verify whether the trend of decreasing energy relative to aqueous TMP is only apparent or if it is physically meaningful and can be attributed to the environment that thymine has in nucleic acids. As can be seen in Tables 2 and 3, we do reproduce the trend; triplet energies decrease upon considering the quantum effects of more adjacent π-stacked thymine nucleobases rather than computing them classically. Analyses of the spin density at the pure singlet (λ = 0) and pure triplet (λ = 1) QM/MM MD simulations almost always show a localized triplet state (Figure 1a). Only in minor cases in the pure triplet simulations, a delocalized electronic structure is found in the CASPT2 electronic-structure computations. This overall localized nature of the triplet thymine in the B-form double helix of DNA agrees with previous works.^8,44,45^ The minor degree of delocalization cannot, therefore, be the factor responsible for the triplet stabilization. Therefore, as stated previously by Allahkaram, Monari and Dumont,^11^ we conclude that the reason for decreasing the triplet energy is the polarization of the adjacent non-excited thymine molecules induced by the triplet electronic density of the excited triplet thymine and the stabilization effect that such polarization has on the excited triplet thymine. The polarization occurs when adjacent nucleobases are included in the QM part of the QM/MM MD simulations and does not happen when they are treated with MM. This finding also suggests that a QM/MM scheme to achieve accurate triplet energies faster than our approach with 4 QM nucleobases would be to use a polarizable force field for the π-stacked nucleobases while keeping the cheaper nonpolarizable force field for the DNA backbone.

**Figure 1:**
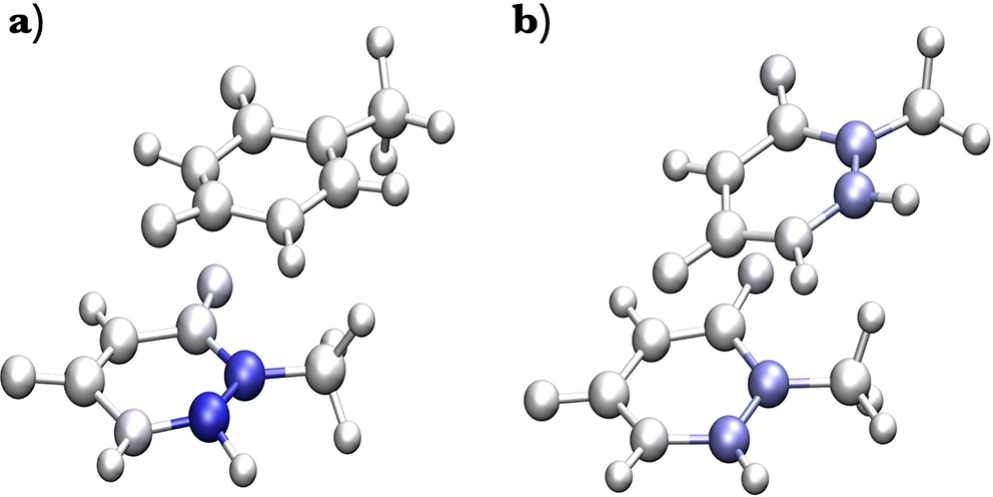
Atomic Mulliken spin populations obtained for representative localized (a) and delocalized (b) triplets of the thymine–thymine dimers obtained with the CASPT2 method on top of QM­(xTB, 2 T)/MM MD conformations. Blue color intensity refers to higher values of the spin (0.0 for white, 1.0 for dark blue; total electronic spin is 2 for a triplet).

## Summary and Conclusions

This study presents a novel methodological approach aimed at calculating precise energies of triplet excited states in DNA, moving beyond existing techniques focusing on internal energies (Δ*U)*. By utilizing a refined free energy perturbation (FEP) method, this work effectively models the transition from a singlet to a triplet state through “alchemical” intermediates with mixed spin states. This approach allows determining singlet-triplet excitation free energies and significantly reduces statistical errors, achieving an accuracy of a few kJ/mol, compared to typical Δ*U* determinations where errors reach tens of kJ/mol. The FEP approach is used with different quantum chemistry levels of theory, including xTB and DFT/B3LYP QM/MM MD simulations and CASPT2-corrected QM/MM MD energy profiles. xTB shows good performance compared to the reference CASPT2 data. The experimental value of 310 kJ/mol for aqueous thymidine monophosphate is reproduced.^43^

The enhanced accuracy of Δ*G* using the developed FEP approach allows for a confident interpretation of the different thymine triplet energies observed experimentally for supercoiled circular DNA (270 kJ/mol)^6,7^ versus aqueous TMP (310 kJ/mol).^6,7,43^ The findings prove the existence of a stabilizing effect of triplet states in DNA, which is attributed to the mutual polarization of the singlet adjacent π-stacked thymine nucleobases with the triplet excited thymine. Excimer interactions contribute only slightly to the triplet stabilization, which is due to the twisted arrangement between adjacent nucleobases forced by the double helix of DNA.

Overall, the developed approach, implemented in the QM^3^ software, has demonstrated to be useful for characterizing triplet electronic states of nucleobases within the biological macromolecular environment of DNA. Such accurate determinations are relevant for advancing cancer treatment approaches. The FEP approach also holds potential for predicting other properties such as free energies of cationic/anionic or charge separation states, with broader applications in fields beyond DNA research, for instance, in materials science.

## $

ProtoQSAR SL, 46980 Paterna, València, Spain
